# Decreased Sulfate Content and Zeta Potential Distinguish Glycosaminoglycans of the Extracellular Matrix of Osteoarthritis Cartilage

**DOI:** 10.3389/fmed.2021.612370

**Published:** 2021-04-29

**Authors:** Rodolfo de Melo Nunes, Virgínia Claudia Carneiro Girão, Pablyana Leila Rodrigues Cunha, Judith Pessoa Andrade Feitosa, Ana Carolina Matias Dinelly Pinto, Francisco Airton Castro Rocha

**Affiliations:** ^1^Department of Internal Medicine, Faculdade de Medicina da Universidade Federal do Ceará, Fortaleza, Brazil; ^2^Department of Morphology, Faculdade de Medicina da Universidade Federal do Ceará, Fortaleza, Brazil; ^3^Departament of Organic and Inorganic Chemistry, Universidade Federal do Ceará, Fortaleza, Brazil

**Keywords:** cartilage, osteoarthritis, zeta potential, sulfate, glycosaminoglycans

## Abstract

We aimed to determine the characteristics that distinguish glycosaminoglycans (GAGs) from osteoarthritis (OA) and normal cartilage and from men and women. Cartilage samples from 30 patients subjected to total joint arthroplasty secondary to OA or fracture (control) were evaluated, and the GAG content (μg/mg dry cartilage) after proteolysis was determined by densitometry, using agarose-gel electrophoresis. Relative percentages of carbon (C), nitrogen (N), and sulfur (S) in GAGs were determined by elemental microanalysis, as well as the zeta potential. Seventeen samples (56.6%) were from patients >70 years old, with 20 (66.6%) from women, and most [20 (66.6%)] were from the hip. The GAG content was similar regardless of patients being >/≤ 70 years old with 96.5 ± 63.5 and 78.5 ± 38.5 μg/mg (*P* = 0.1917), respectively. GAG content was higher in women as compared to men, with 89.5 ± 34.3 and 51.8 ± 13.3 μg/mg, respectively (*P* = 0.0022), as well as in OA than fracture samples, with 98.4 ± 63.5 and 63.6 ± 19.6 μg/mg, respectively (*P* = 0.0355). The GAG extracted from the cartilage of patients >70 years old had increase in N, and there were no gender differences regarding GAG elemental analysis. GAG from OA had a highly significant (*P* = 0.0005) decrease in S% (1.79% ± 0.25%), as compared to fracture samples (2.3% ± 0.19%), with an associated and significant (*P* = 0.0001) reduction of the zeta potential in the OA group. This is the first report of a reduced S content in GAG from OA patients, which is associated with a reduced zeta potential.

## Introduction

There is a great need for accurate, reproducible biomarkers to be used in the management of osteoarthritis (OA). Without adequate parameters, it is virtually impossible to evaluate interventions to treat OA patients. Numerous attempts to quantitate soluble biomarkers or to use imaging techniques have failed to provide those most needed biomarkers (1). When subjected to OA damage, inadequate proliferation and osteophyte formation in the joint are followed by complete disruption and erosion of the cartilage leading to bare areas exposing the underlying subchondral bone. Proliferative rather than degenerative changes occurring in OA lead to osteophyte formation (2). It thus might well be that qualitative rather than quantitative changes will help distinguish OA from non-OA (normal) cartilage. Type II collagen and chondroitin sulfate (CS)–rich proteoglycans represent the major organic components of the extracellular joint cartilage matrix. Assembly of type II collagen and proteoglycans is essential for cartilage function, a highly hydrated structure that is frequently subjected to reversible deformation. Also, the number of glycosaminoglycan (GAG) chains attached to the protein backbone of proteoglycans and their aggregating pattern are relevant to cartilage homeostasis (3, 4). Modifications of GAG have been found in the cartilage obtained from OA patients. The molar mass (MM) of hyaluronan, which is the main GAG in joint cartilage, was shown to be altered in areas of more severely damaged cartilage of knee OA patients (5, 6). Also, CS extracted from the cartilage of areas most severely affected by OA was shown to display reduced MM (7, 8).

In addition to the biological and biochemical characteristics, the electric charge of extracellular components of matrix cartilage is also relevant to homeostasis (9). The presence of both carboxylate (COO–) and sulfate (OSO3–) groups linked to the GAG attached to the protein core of proteoglycans provides a negative surface charge that is crucial to cartilage function, particularly during weight-bearing deformation (10). The zeta potential is a parameter that measures the surface charge of nanoparticles/macromolecules that can be either negative or positive, depending on the predominant electrical charge. The repulsion between surfaces of similar charge is responsible for the stability of a dispersion. When the zeta potential is reduced, compounds dispersed in a matrix have a tendency to aggregate (11). In this regard, the negatively charged groups present in GAG from joint cartilage account for its negative zeta potential. It has been shown that a zeta potential of 0 ± 10 mV compromises the stability of a polysaccharide in a solution, which then exhibits a tendency to flocculate (12). Maintenance of GAG stability may impact cell function as it was shown that *in vitro* chondrocyte growth is optimized in negatively charged rather than in neutral hydrogel matrices (13). We performed an elemental microanalysis of GAG extracted from the cartilage of patients subjected to arthroplasty either secondary to OA or that sustained a fracture (control) in an attempt to detect differences between those groups.

## Materials and Methods

### Materials

Unless otherwise stated, all reagents were purchased from Sigma–Aldrich do Brasil S.A., São Paulo, Brazil.

### Collection of Human Cartilage Samples

Thirty cartilage samples from patients subjected to total joint arthroplasty in the Hospital Universitário Walter Cantídio, Fortaleza-CE, Brazil, secondary to OA or fracture (control) were collected.

### Inclusion Criteria

Fifty- to 80-year-old patients subjected to total hip or shoulder arthroplasty secondary to OA or fracture, according to the clinical files, with body mass index <35 kg/m^2^; a written informed consent was signed prior to collection of the material, as per the Brazilian rules of human experimentation.

### Exclusion Criteria

refusal of the patient to participate at any time, presence of diabetes or a specific acute or chronic inflammatory arthropathy but OA. Patients who died following remote postsurgical period had the material discarded.

Prior to collecting samples, the clinical history of each patient and radiographies of the joint that would undergo arthroplasty were reevaluated by a senior rheumatologist (F.A.C.R.) both to confirm OA diagnosis and to exclude OA or other arthropathy in patients with a fracture diagnosis. GAGs were extracted within <3 h postsurgery and analyzed as described above. The protocol was approved by our local Ethics Committee on Human Research (protocol 090.12.08) that follows the rules of the Comitê Nacional de Ética em Pesquisa, which is the Brazilian Official Committee for Ethics in Human Research. All patients signed a written informed consent prior to any procedure.

### GAG Extraction

Cartilage samples were weighed after overnight drying (80°C) and stored in acetone. Proteolysis of this material was done by incubating 1 mg with 20 μL of a 0.4% wt/vol suspension of PROLAV 750™ (Prozyn, São Paulo, Brazil) in Tris–HCl/NaCl 50/150 mmol/L buffer (pH 8.0) for 48 h, at 56°C. Subsequently, the NaCl concentration was corrected to 1.0 mol/L, and the mixture was kept at 37°C during 30 min. The remaining proteins were precipitated with trichloroacetic acid to a final concentration of 10% wt/vol and centrifuged (10,000 *g* for 15 min at 25°C). GAG was precipitated from the supernatant with two volumes of ethanol, followed by an overnight incubation at 4°C and centrifugation (10,000 *g* for 15 min at 15°C). The precipitated material was dissolved in 20 μL of distilled water. Protein content in the debris was considered negligible as it was undetected even using a NanoDrop apparatus.

### GAG Quantification

The GAG extract was separated on a 0.6% wt/vol agarose-gel electrophoresis in diaminopropane–acetate buffer (50 mmol/L, pH 9.0). The GAG was fixed in the gel through immersion in a 0.1% wt/vol cetyl-trimethylammoniun bromide solution for 2 h. The gel was dried and stained with 0.1% wt/vol toluidine blue (in acetic acid:water:ethanol 1:49:50). For comparison, C4S, C6S, and heparan sulfate standards were subjected to the same protocol (14). Quantification was made by densitometry (525 nm). Data are expressed as μg CS/mg of dried cartilage.

### Elemental Analysis of GAG

The relative percentages of carbon (C), nitrogen (N), and sulfur (S) were determined by elemental microanalysis in a Carlo Erba EA 1108 micro. The sulfate content was calculated from S% by a previously proposed equation (15). This strategy subjects the sample to combustion in pure oxygen atmosphere, and the expelled gases are detected and semiquantified using a thermal conductivity detector. Thus, the oxygen content cannot be determined.

### Determination of the Zeta Potential (Pζ)

Zeta potential and conductivity were measured by a Zetasizer Nano ZS90 instrument (Malvern Instruments Ltd., Worcestershire, United Kingdom) with an λ = 633-nm laser detector with a 17° detection angle, at 25°C. GAG samples (50 μg/mL in deionized water) were swollen in deionized water to the equilibrium state and ground into small particles. After drying in a vacuum oven for 24 h, the particles were weighed, diluted in 1 mL deionized water, and measured.

### Statistics

Results are presented as means ± SD for GAG concentration and medians [interquartile range (IQR)] for percentage of elements and evaluated using Student *t*-test and Kruskal–Wallis test, respectively; *P* < 0.05 was considered as significant.

## Results

### Clinical Demographics

A total of 30 cartilage samples were collected. There were 17 samples (56.6%) from patients >70 years old, with 20 (66.6%) collected from women, and most [20 (66.6%)] were hip samples (Table 1). According to the clinical history, all fractures occurred after falls with minimal trauma.

**Table 1:** Clinical features of patients subjected to arthroplasty secondary to Osteoarthritis (OA) or fracture (control).

	**Groups**	**Fracture**	**OA**
Age (Mean± SD)		67 ± 5	67 ± 4
Age range	≤ 70	6	7
	> 70	10	7
Gender	Female	10	10
	Male	6	4
Joint	Hip	10	10
	Shoulder	6	4

### Quantification of the GAG Extracted From the Articular Cartilage

**Figure 1:**
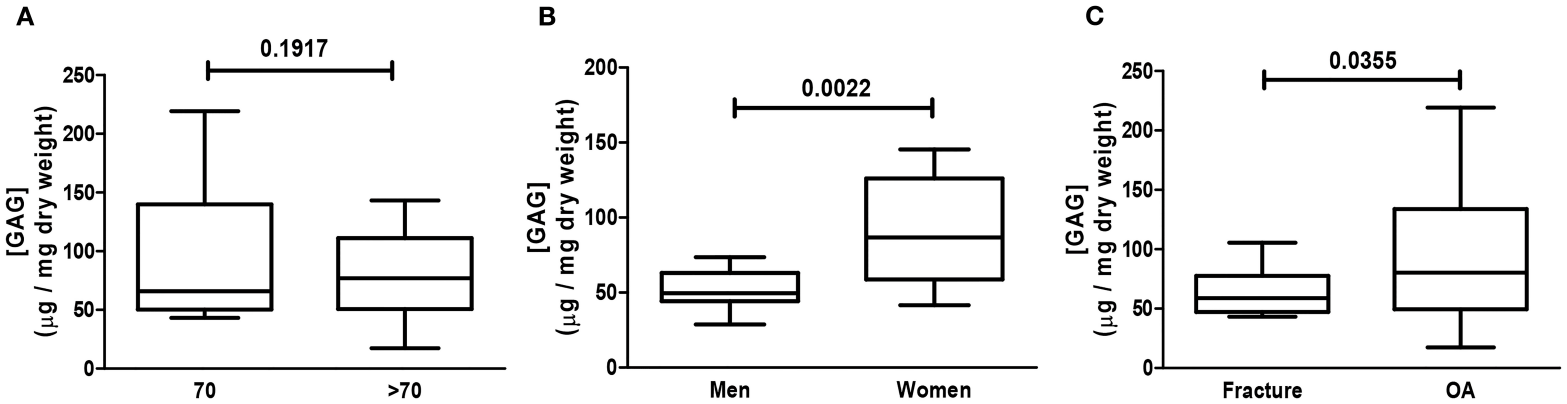
Assessment of glycosaminoglycan (GAG) content. Human cartilage samples from patients subjected to arthroplasty secondary to OA or fracture were assessed for GAG content. Data represent mean ± SEM of GAG content (μg/mg) of dried cartilage as follows: patients >/≤ 70 years old **(A)**, gender **(B)**, and OA and fracture **(C)** of at least *n* = 6/group; Student *t*-test.

The relative GAG content in analyzed samples was in the range of 50–85 wt%. Figure 1 illustrates that the GAG content relative to the dried cartilage weight was similar regardless of patients being >/≤ 70 years old (a); regarding gender, the relative GAG content was significantly higher in samples from women (b); there was also a significantly higher increase in the relative GAG content in samples from OA patients as compared to samples from patients who sustained a fracture (c).

### Elemental Analysis of GAG

**Figure 2:**
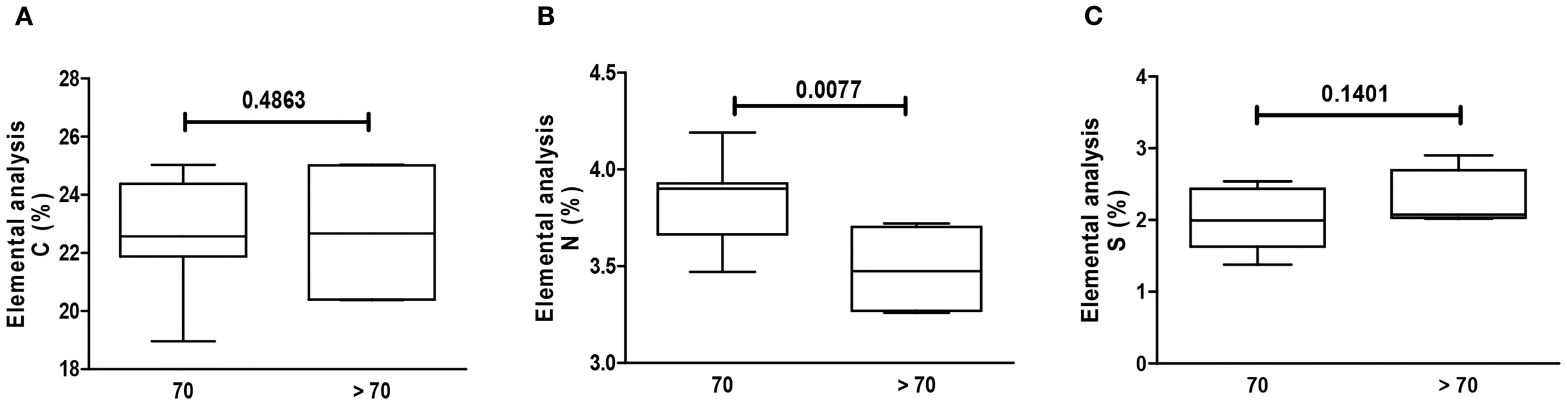
Elemental analysis of GAG. Elemental analysis of GAG from human cartilage samples of patients >/≤ 70 years old subjected to arthroplasty secondary to OA or fracture. Data represent medians (IQR) of relative percentage of carbon (C), nitrogen (N), hydrogen (H), and sulfate (S) of at least *n* = 6/group; Kruskal–Wallis test.

**Figure 3:**
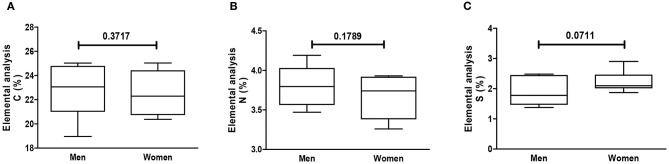
Elemental analysis of GAG per gender. Elemental analysis of GAG from human cartilage samples of patients subjected to arthroplasty secondary to OA or fracture patients. Data represent medians (IQR) of relative percentage of carbon (C), nitrogen (N), hydrogen (H), and sulfate (S) of at least *n* = 6/group; Kruskal–Wallis test.

**Figure 4:**
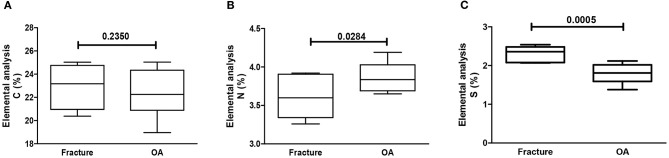
Elemental analysis of GAG. Elemental analysis of GAG from human cartilage samples of patients subjected to arthroplasty secondary to OA or fracture. Data represent medians (IQR) of relative percentage of carbon (C), nitrogen (N), hydrogen (H), and sulfate (S) of at least *n* = 6/group; Kruskal–Wallis test.

Using CS as reference (chemical formula C_13_H_21_NO_15_S), the relative C, N, and S percentages are roughly 34, 3, and 7 wt%, respectively. However, the observed percentage values of C (circa 23 wt%) and S (circa 2 wt%) were smaller than the predicted theoretical values, particularly regarding S content. Elemental analysis showed that GAG from the cartilage of patients >70 years old had a significant decrease in N, as compared to patients ≤70 years old (Figure 2). There were no differences in C, N, or S relative content regarding gender (Figure 3). Remarkably, the GAG extracted from the cartilage of patients with OA had a highly significant decrease in the relative S content, as compared to samples obtained from patients who sustained a fracture (Figure 4). There was also a slightly higher N relative content in GAG samples from OA patients, which reached statistical significance, probably secondary to the relative reduction of the S content in that group (Figure 4).

### Analysis of the Zeta Potential (Pζ) of GAG

**Figure 5:**
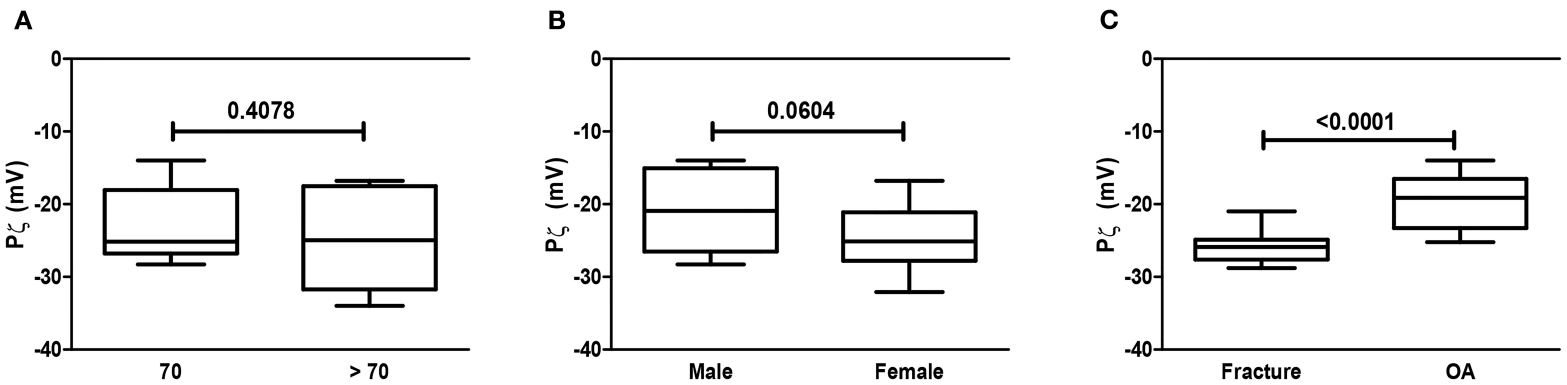
Analysis of the zeta potential (Pz). Analysis of the Pz of GAG from human cartilage samples of patients subjected to arthroplasty secondary to OA or fracture. Data are expressed as mean ± SEM of zeta potential (mV) considering age (>/≤ 70 years old) **(A)**, gender **(B)**, and **(C)** OA/fracture of at least *n* = 6/group; Student *t*-test.

Figure 5 illustrates the analysis of the zeta potential considering age, gender, and disease variation. As expected, all samples had a negative zeta potential, which varied within a −19 to −26 mV range. The zeta potential of GAG samples from the articular cartilage of patients >/≤ 70 years old was similar (Figure 5A); regarding gender, there was a slight variation as cartilage samples obtained from women had a trend toward a higher modulus (−26 mV), meaning being more negative, although not reaching statistical significance, when compared to GAG samples from men (Figure 5B); finally, there was a remarkable significant reduction of the zeta potential in GAG samples collected from the cartilage of OA patients as compared to those from patients who sustained a fracture (Figure 5C).

## Discussion

The present data describe a decrease in the sulfate content and a correspondent decrease in the zeta potential of GAG extracted from the cartilage of joints affected by OA. There is also an increase in the relative GAG content in samples from OA patients, as compared to those from fracture (control) patients.

Both GAG content and integrity are crucial to the aggrecan role in cartilage physiology. It was reported that CS obtained from OA cartilage exhibits structural alterations, meaning different length and sulfation patterns, which may impact cartilage function (7, 8). Our data reinforce those findings to suggest that qualitative changes reflect cartilage damage in OA joints. Integrity of GAG molecules is crucial to provide deformability of the cartilage particularly during weight-bearing. Additionally, GAGs are able to specifically bind to cytokines and growth factors, triggering intracellular signaling. Thus, structural modifications of the GAG structure may impact cellular responses, thus altering the function of cartilage and synovial cells (16–18).

Increased GAG content in OA cartilage has been previously shown and may illustrate the initial proliferative response of chondrocytes, as part of a repairing process. However, subjected to an OA inflammatory milieu, the “osteoarthritic chondrocytes” not only lose capacity to synthesize normal GAG but also fail to replace normal cartilage. In later stages, joint erosion occurs, with areas of denuded cartilage and exposure of the subchondral bone (4, 5). In fact, our OA samples were from patients with end-stage disease, and all possible remaining cartilage was collected. Gross macroscopic evaluation does not always allow discriminating normal from damaged cartilage. Although we cannot rule out some remaining areas of normal cartilage in the OA samples, data were treated as one sample for each patient. Cartilage from dogs subjected to an experimental OA model had increased amount of proteoglycans, as compared to controls (6). Also, joint cartilage collected from humans subjected to hip arthroplasty secondary to OA was shown to have an increase in GAG content, as compared to cartilage collected from patients with fracture of that joint, used as control (19). Notwithstanding, analysis of magnetic resonance imaging of OA joints revealed an increase in matrix production in patients with recently developed OA, an aspect that was regarded as part of a repair mechanism (20, 21). Although still seen as a degenerative disease, characteristic imaging findings in the OA joint reveal sclerosis of the subchondral bone and osteophyte formation, which gives an impression of *de novo* remodeling. In keeping with those data, the relative increase in the GAG content found in OA samples may be secondary to a proliferative, although inadequate, process happening in the affected joint.

We are not aware of previous studies showing elemental analysis of GAG isolated from the cartilage of human joints. However, at least regarding S content, a similar order of relative percentage (0.7–1.3%) was found in cartilage obtained from dogs (22). Our samples were processed just after surgical removal, aiming to avoid any possible alterations that could be due to postmortem modifications or freeze-thawing issues. There was an increase in the relative GAG content in dried cartilage seen in samples from women, which could be linked to a higher number of women in the OA group. Samples from OA cartilage had a remarkable significant decrease in the S% content as compared to control samples of patients with similar age range. Indeed, all but one of the GAG samples from the OA group had a relative S% content in the lowest value found in fracture patients. Increased thickness of OA cartilage using delayed gadolinium-enhanced magnetic resonance was associated with increased swelling secondary to a decrease in GAG content (23). In keeping with our present data, using micro–X-ray fluorescence, it was shown that the deep zones of OA cartilage have a decrease in elemental S, which was associated with a decrease in GAG staining (24). The modulus of the zeta potential of the GAG from OA samples was significantly reduced, meaning a reduction in the negative charge of the polysaccharides probably secondary to the reduced S% relative content. We are not aware of previous studies focusing on the relevance of GAG charge to cartilage physiology. Sulfation of GAG is responsible for the negative charge of those molecules. After a compressive force applied to the cartilage, the repulsion between adjacent negatively charged GAG molecules allows the entry of water providing adequate cartilage hydration, which is crucial to a healthy joint (25). Although there is a positive association of the zeta potential and the stability of small particles, reducing its tendency to aggregate (13, 26, 27), there are no previous studies on the stability of GAG, let alone the relevance of the zeta potential of those molecules. However, it is reasonable to admit that a normal sulfation pattern contributes to the physiology of polysaccharides in the extracellular cartilage matrix. In this case, a reduced charge does also compromise hydration of the cartilage (28, 29).

Biomarkers to be used in clinical practice are an unmet need in OA (1). Our data show that a decrease in sulfation is associated with a correspondent reduction in the zeta potential of GAG collected from OA cartilage. Current imaging studies can be designed to deliver markers able to quantitate the sulfate content or the charge of GAG in the joint cartilage. Mapping those alterations may provide semiquantitative imaging useful to evaluate interventions to modify the disease course in OA patients.

There are some limitations to our study, including time sampling. However, as mentioned previously, all material was processed within <3 h postsurgery. Additionally, GAGs are very stable and probably would not be affected by processing. One may also argue that our samples represent solely end-stage OA joint disease. Collecting enough material from living humans is very hard, and ethical rules do apply. Considering that we analyzed the whole joint, we probably saw the predominant parameter in all remaining cartilage. However, it remains to be shown if such data are reproduced in less severely affected joints. Another limitation is the low number of samples, particularly those from men, limiting gender analysis. We also restricted our samples to the hip and shoulders, and the low numbers did not allow us to compare possible differences regarding specific joints. Although knee OA is more prevalent than hip OA (30), knee fractures that lead to joint replacement are rare, making it difficult to have a suitable non-OA knee control. We also cannot completely rule out subclinical OA changes in fracture (control) samples. However, our combined clinical and imaging rheumatologic and orthopedic evaluations suggest that an OA diagnosis in the fracture group is unlikely.

In summary, we demonstrate that cartilage from OA samples displays a relative increase in the CS content. We also show that GAGs from the extracellular matrix of joints affected by OA have a decrease in sulfate content, which is associated with a decrease of the zeta potential of those polysaccharides. The possible relevance to the pathophysiology of this disease, as well as utility as a biomarker, warrants further investigation.

## Data Availability Statement

The raw data supporting the conclusions of this article will be made available by the authors, without undue reservation.

## Ethics Statement

The studies involving human participants were reviewed and approved by Conselho de Ética do HUWC-UFC (Protocol number 090.12.08). The patients/participants provided their written informed consent to participate in this study.

## Author Contributions

FR and JF conceived the protocol. RN, AP, VG, PC, and FR performed experiments and sample collection. RN, JF, and FR performed data analysis. All authors wrote, revised, and approved final manuscript.

## Conflict of Interest

The authors declare that the research was conducted in the absence of any commercial or financial relationships that could be construed as a potential conflict of interest. The reviewer JF is currently organizing a Research Topic with the author FR.
